# Cardiovascular Remodeling in Chronic Mineralocorticoid Excess

**DOI:** 10.7759/cureus.40753

**Published:** 2023-06-21

**Authors:** Pradnya Brijmohan Bhattad, Mazen Roumia

**Affiliations:** 1 Cardiovascular Medicine, Saint Vincent Hospital, UMass Chan Medical School, Worcester, USA

**Keywords:** conn syndrome, hypokalemic metabolic alkalosis, resistant hypertension, diastolic dysfunction, heart failure with preserved ejection fraction, primary aldosteronism, left ventricular dysfunction

## Abstract

Primary hyperaldosteronism typically leads to resistant hypertension, hypokalemia, and metabolic alkalosis. Excess aldosterone secretion by the adrenal glands may lead to heart failure with preserved ejection fraction. Potassium-sparing diuretics and aldosterone antagonists directed to lower excess aldosterone levels may help treat the associated heart failure and lead to control of blood pressure, resulting in improved outcomes.

We report a case of a 55-year-old male with poorly controlled hypertension and newly symptomatic heart failure with preserved ejection fraction in the setting of excess aldosterone activity and an adrenal adenoma suggesting primary aldosteronism-induced diastolic heart failure.

The biochemical evaluation revealed elevated plasma aldosterone concentrations with low plasma renin activity, diuretic-induced hypokalemia, and metabolic alkalosis. A progressively enlarging left adrenal adenoma was found on abdominal imaging along with resistant hypertension despite the use of multiple antihypertensive medications. Medical management targeted to lower excess aldosterone levels with the use of aldosterone antagonists helped us achieve better blood pressure control and resolution of symptoms of diastolic dysfunction. Treating the underlying pathology helped us improve overt heart failure and may suggest that goal-directed therapy towards the inciting factors may potentially lead to a path to reverse the heart failure symptoms clinically.

## Introduction

Primary aldosteronism is also known as Conn’s syndrome and is characterized by excess secretion of aldosterone, either due to bilateral adrenal hyperplasia or an aldosterone-secreting adenoma [1]. Patients presenting with hypertension, usually difficult to treat or poorly controlled, hypokalemia, and alkalosis should be suspected to have hyperaldosteronism [2-4]. Some individuals may not have spontaneous hypokalemia but develop severe hypokalemia on the administration of diuretics. Plasma renin levels are often low. 24-hour urine aldosterone levels are often greater than 40 mEq [1,3-6]. Aldosterone-secreting adenomas may be treated by unilateral adrenalectomy. Medical management involves the control of hypertension with antihypertensive agents and the use of potassium-sparing diuretics such as spironolactone and amiloride [3,4,7-10]. Excess aldosterone in primary hyperaldosteronism leads to decreased renal reabsorption of potassium and hydrogen ions, resulting in hypokalemia and metabolic alkalosis; increased renal sodium reabsorption leads to increased blood volume with hypertension, resulting in low renin and low angiotensin II levels [6,10-13].

## Case presentation

A 55-year-old male with a past medical history of long-standing poorly controlled hypertension, dyslipidemia, and an unspecified adrenal adenoma presented with shortness of breath, bilateral lower extremity edema, generalized weakness, fatigue, lower extremity cramping, and weight gain of three weeks duration. He reported dyspnea at rest and orthopnea on presentation. He gained 20-30 pounds in the three weeks before the presentation. He complained of mild generalized abdominal pain and worsening abdominal distention in the preceding three weeks. Three weeks before presentation, he was seen in the outpatient clinic and started on Furosemide. 

A review of systems was otherwise negative. He reported that he was diagnosed with an adrenal adenoma several years ago but lost the opportunity to follow up. He denied any use of tobacco, alcohol, and illicit drugs. He was adopted, and hence, his family history was unknown. On initial exam in the emergency room, his blood pressure was 184/102 mmHg with a pulse of 76 bpm; other vitals were stable. On auscultation, bibasilar crackles were audible. The abdomen was severely distended but non-tender to palpation. 3+ bilateral lower extremity pitting edema extending up to both knees was noted on physical examination. Refer to Table 1 below for the biochemical workup and findings during the admission.

**Table 1:** Laboratory test evaluation at the time of hospitalization

Test	Result	Reference range	
Pro B-Type natriuretic peptide (pro-BNP)	1119	Less than 125pg/mL	
Serum Potassium	2.6	3.5-5.1 mmol/L	
Serum Bicarbonate	34	19-32 mmol/L	
Anion Gap	12	2-11	
Serum Total Bilirubin	2.4	0.3-1.2 mg/dL	
Alanine Transaminase	47	0-40 U/L	
Aspartate Transaminase	46	0-39 U/L	
Alkaline Phosphatase	62	39-117 U/L	
Arterial pH on room air	7.52	7.35-7.45	
Arterial Bicarbonate	39.1	22-26 mmol/L	
Arterial PCO2 on room air	47.7	35-45 mmHg	
Arterial PO2 on room air	50	82-92 mmHg	
Serum Phosphorous	3.5	2.5-4.5 mg/dL	
Serum Magnesium	2.1	1.5-2.5 mg/dL	
AM Cortisol	9.8	6-18.4 ug/dL	
Thyroid Stimulating Hormone	2.96	0.34-5.60 uIU/mL	
Plasma Renin Activity	0.06	0.25-5.82 ng/ml/h	
Plasma Aldosterone	19	Upright 8:00-10:00 am < or = 28 ng/dL	
Concentration at 04:00 am			
		Upright 4:00-6:00 pm < or =21 ng/dL	
		Supine 8:00am-10:00 am 3-16 ng/dL	
24-hour Urine Aldosterone	22.7	2.3-21.0 mcg/24hours	
24-hour Urine Creatinine	2.13	0.50-2.15g/24hours	
Serum Sodium	142	135-145 mmol/L	
Serum Chloride	96	96-112 mmol/L	
Serum Creatinine	0.85	0.50-1.35 mg/dL	

**Figure 1:**
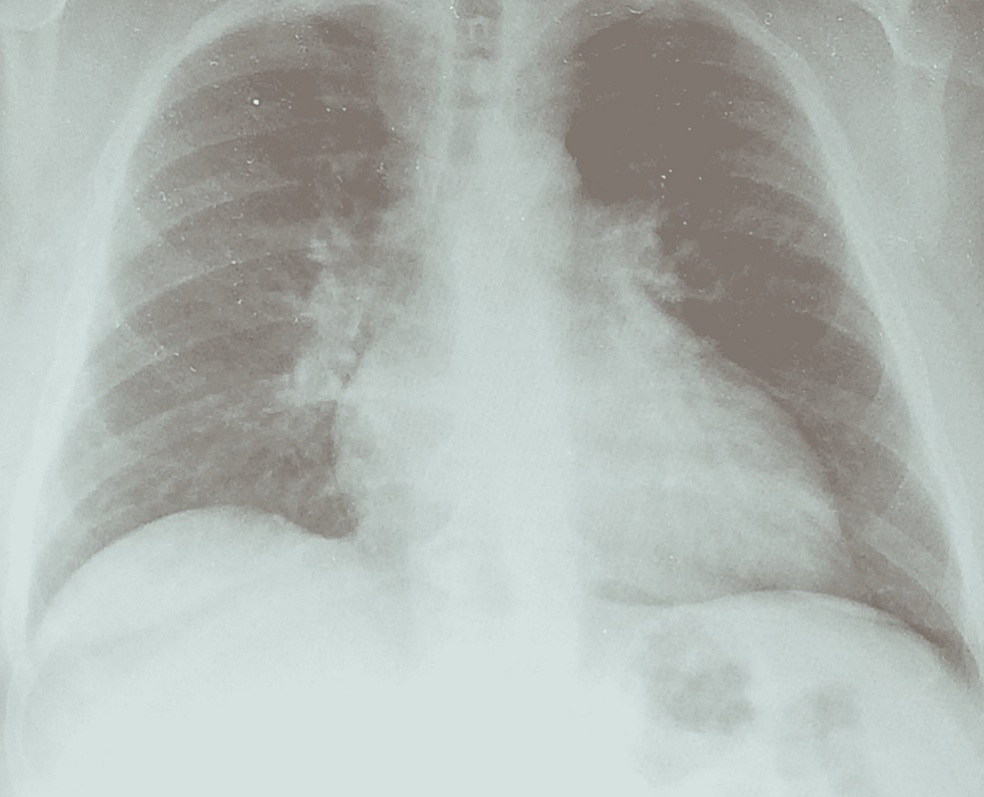
Chest X-ray showing mild pulmonary edema

**Figure 2:**
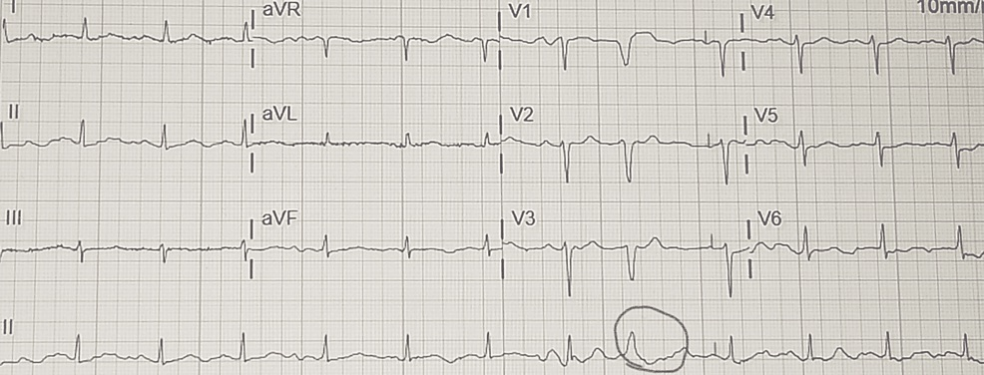
An ECG showing normal sinus rhythm with ventricular premature complexes and left atrial enlargement ECG: Electrocardiogram

A computed tomography (CT) imaging of the abdomen and pelvis with contrast revealed a 3.6 cm left adrenal nodule characterized as an adenoma, anasarca, ground-glass bibasilar lung opacities, and hepatic steatosis. The left adrenal nodule measured 2.8 cm in size ten years before this admission and 2.3 cm fourteen years before this admission on review of previous CT imaging of the abdomen, indicating growth of the nodule over time. A chest X-ray revealed mild pulmonary edema with bibasilar atelectasis (Figure 1). An electrocardiogram (ECG) revealed normal sinus rhythm with ventricular premature complexes, a borderline prolonged PR interval, a prolonged QT interval, and left atrial enlargement (Figure 2).

**Figure 3:**
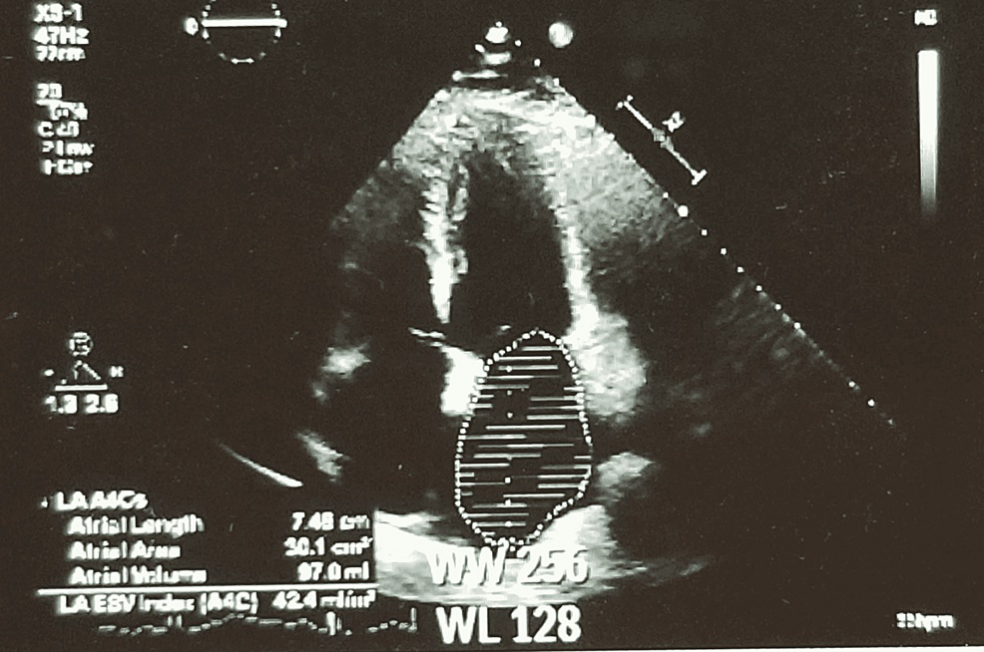
2D Echocardiogram demonstrating moderately dilated left atrium with a left atrial volume index of 42.4ml/m^2

A complete two-dimensional transthoracic echocardiogram with contrast revealed normal left and right ventricular systolic function. Left ventricular ejection fraction was 55% to 60%, and no wall motion abnormality was detected; grade 3 diastolic dysfunction (restrictive with an E/A ratio of 2.2), moderately dilated left atrium with a left atrial volume index of 42.4ml/m^2, peak tricuspid regurgitation jet velocity was 2.91m/s, right ventricular systolic pressure was 45 to 50 mmHg with a dilated inferior vena cava. Thus, the echocardiogram revealed left atrial volume enlargement and elevated right heart pressures consistent with advanced diastolic dysfunction (Figure 3). 

On admission, potassium was aggressively replaced. Furosemide was not administered immediately on admission due to severe hypokalemia.

Instead, he was started on spironolactone (25 mg by mouth daily) to treat volume overload and hypokalemia. Once the hypokalemia improved, he was started on aggressive intravenous diuresis with furosemide along with potassium supplementation. Eventually, he was switched to furosemide by mouth in combination with spironolactone for continued diuresis. After clinical improvement and the achievement of a euvolemic state with stable electrolytes, he was discharged home on furosemide, spironolactone, and potassium supplementation. He required clonidine, labetalol, amlodipine, and lisinopril for adequate control of resistant hypertension upon discharge. On outpatient follow-up in one week, his dyspnea and orthopnea had resolved completely, he lost about 40 pounds of weight, and he reported significant improvement in his lower extremity edema with a resolution of abdominal discomfort and distension. Based on his treatment preferences, any invasive evaluation was deferred. He was closely followed up further in the outpatient setting and was referred to endocrinology as well for the follow-up of the left adrenal adenoma.

## Discussion

Our patient developed diuretic-induced hypokalemia, metabolic alkalosis, and refractory hypertension despite being on three antihypertensive medications. He was previously never treated with diuretics and was started on furosemide 3 to 4 weeks before presentation, after which he developed severe hypokalemia consistent with diuretic-induced hypokalemia. The constellation of findings of hypokalemic metabolic alkalosis with resistant hypertension in the setting of a known adrenal adenoma suggests primary aldosteronism. Analyzing his biochemical workup reveals a plasma aldosterone concentration to plasma renin activity (PAC/PRA) ratio of 316.66, which is another strong clue to primary aldosteronism. He presented with acutely decompensated congestive heart failure with preserved ejection fraction. His advanced diastolic dysfunction was likely contributed by the effects of excess aldosterone from primary aldosteronism, leading to uncontrolled hypertension. The use of an aldosterone antagonist, spironolactone, resulted in better blood pressure control, resolution of his symptoms consistent with diastolic dysfunction, along with maintenance of stable serum potassium levels. 

A morning PAC/PRA ratio greater than 20 with a PAC greater than 15ng/dL is suggestive of primary hyperaldosteronism [4,10,11,13]. The diagnosis of primary aldosteronism may be confirmed by an aldosterone suppression test. This aldosterone suppression test may be omitted in cases of PAC greater than 30ng/dL or undetectable PRA, as these findings are unlikely from other causes [6,10-14].

Primary hyperaldosteronism has been linked to an elevated risk of congestive heart failure based on a review of previous studies. A prompt diagnosis of primary hyperaldosteronism and targeted therapy may be associated with improved outcomes in these patients. There is currently not enough evidence to identify any patterns of association between aldosterone-producing adrenal adenomas and new-onset congestive heart failure [14,15]. 

## Conclusions

Primary hyperaldosteronism is characterized by hypertension, an elevated PAC/PRA ratio, and hypokalemic metabolic alkalosis. CT of the adrenal glands may help distinguish between bilateral adrenal hyperplasia and adrenal adenoma. Medical therapy with aldosterone antagonists such as spironolactone or eplerenone is preferred in bilateral adrenal hyperplasia and in patients with unilateral adrenal adenoma who are poor surgical candidates or who refuse surgery, whereas unilateral adrenalectomy is preferred for a unilateral adrenal adenoma. Goal-directed therapy towards the inciting factor may help treat heart failure in such presentations.
